# The Elevated Expressions of Anti-lipopolysaccharide Factors After Priming Stimulation Confer Lastingly Humoral Protection in Crab *Eriocheir sinensis*

**DOI:** 10.3389/fimmu.2021.757434

**Published:** 2021-12-08

**Authors:** Weilin Wang, Yan Li, Siqi Fan, Xingye Lian, Wanqing Cao, Xiaorui Song, Qilin Yi, Lingling Wang, Linsheng Song

**Affiliations:** ^1^ Liaoning Key Laboratory of Marine Animal Immunology and Disease Control, Dalian Ocean University, Dalian, China; ^2^ Liaoning Key Laboratory of Marine Animal Immunology, Dalian Ocean University, Dalian, China; ^3^ Dalian Key Laboratory of Aquatic Animal Diseases Prevention and Control, Dalian Ocean University, Dalian, China; ^4^ Functional Laboratory of Marine Fisheries Science and Food Production Process, Qingdao National Laboratory for Marine Science and Technology, Qingdao, China

**Keywords:** immune memory, humoral immune protection, Chinese mitten crab, anti-lipopolysaccharide factor (ALF), TLR signal

## Abstract

Evidence of immune memory in invertebrates (immune priming) has accumulated in various organisms, and both cellular and humoral immune reactions are speculated to be involved in immune priming. However, there is a lack of understanding of the molecular mechanisms involved. In the present study, the protective effect of primed haemolymph was further validated by the increased survival rate of naïve crabs receiving a transfusion of primed haemolymph. By proteomic analysis, there were 474 proteins identified from the primed haemolymph, and most of them were functionally annotated in transport and metabolism classes. A total of 70 proteins were found to be differentially expressed in haemolymph at 12 hours and 7 days after priming stimulation with *Aeromonas hydrophila*, among which anti-lipopolysaccharide factor 1 (*Es*ALF-1) and 3 (*Es*ALF-3) were identified as the most significant (*p* < 0.05). After being challenged with *A. hydrophila*, *Es*ALF-1 and *Es*ALF-3 were highly expressed at both mRNA (in haemocytes) and protein (in haemolymph) levels compared with blank crabs, and the mRNA expressions of components in the *Es*TLR1-*Es*Myd88-*Es*Pelle-*Es*ALF pathway also increased significantly (*p* < 0.05). The *Es*ALF-3 and *Es*Myd88 were even significantly higher expressed in response to the second *A*. *hydrophila* challenge, but their expressions all decreased (*p* < 0.05) when *Es*TLR1 was knocked down by RNAi. After the naïve crabs received an injection with the recombinant protein of *Es*ALF-1 (r*Es*ALF-1) or *Es*ALF-3 (r*Es*ALF-3), their survival rate increased significantly (*p* < 0.05) upon *A*. *hydrophila* stimulation. In contrast, the survival rate of the primed crabs reduced significantly (*p* < 0.05) after they received an injection with the antibody of *Es*ALF-1 or *Es*ALF-3. The enhanced expressions of *E*sALF-1 and *Es*ALF-3 after *A*. *hydrophila*p riming stimulation could sustain for four weeks. All the results suggested that the *Es*TLR1-mediated productions of *Es*ALF-1 and *Es*ALF-3 in haemolymph played an indispensable role in the month-long humoral immune protection induced by *A*. *hydrophila*, which provides solid evidence of immune priming in crabs and a valuable reference for further understanding immune memory in invertebrates.

## Introduction

The passive immune protection of sera from pre-exposed individuals was uncovered and applied in humans a hundred years ago, leading to subsequent study of immune memory and the development of vaccines. Various kinds of vaccines have been widely used in vertebrates to induce immune protection including humoral and cellular immune responses. Elevated specific antibodies and related complement components in serum after pre-infection or vaccination provide long-lasting enhanced protection from re-infection (1). This convalescent plasma can even confer enhanced resistance in naïve individuals against pathogens when it is transferred into recipients, which is used as an emergent clinical therapy in humans for fatal infectious diseases (2, 3).

Enhanced immune protection after pre-infection is also increasingly reported in mollusks, insects, and crustaceans, indicating the existence of immune memory (named immune priming) or immune enhancement in invertebrates (4–6). In these animals, the immune defense capacity and survival rate increase significantly when they encounter the same pathogen for the second time. The immune priming response has been summarized as having the following characteristics: (i) specific immune resistance, (ii) long-lasting protection, and (iii) a biphasic immune response (increasing after the first challenge and returning to basal levels and increasing again to a greater degree after the second challenge) (7). Even though it did not exhibit specificity and memory, immune enhancement resulting from sustained immune response after a first challenge could protect against a second challenge. Both the humoral and cell-mediated immune responses are found to be involved in enhanced immune protection or immune priming. For instance, prior pathogen exposure brought about faster bacterial clearance and a significantly higher survival rate in the insect *Bombus terrestris* upon the second exposure (8). The priming with a sub-lethal dose of *Streptococcus pneumonia* could protect insect *Drosophila* from a lethal second challenge, and the phagocytosis activity of haemocytes was found to play an indispensable role in improved survival rates (9). Enhanced cellular immune responses (phagocytic activity and hematopoiesis) and humoral immune reactions (anti-bacterial and anti-oxidant activities) have also been reported in the scallop *Chlamys farreri* and the oysters *Crassostrea gigas* after a short-term pre-exposure with related pathogens *Listonella anguillarum* or *Vibrio splendidus* (10–13). A number of effector molecules and immune related signal pathways have been suggested to be involved in these humoral and cellular protective responses. For example, the antibacterial peptide biomphalysin was found to be indispensable for the development of immune enhancement in snail *Biomphalaria glabrata* (14). The Toll pathway was found to be required for *S. pneumonia* induced immune priming in *Drosophila* (9). The TLR signaling pathway and antibacterial-related molecules proline-rich protein were also significantly induced in oysters after *V. splendidus* priming stimulation (12). It has been suggested that the activation of the Toll signal pathway and production of antibacterial molecules might play important roles in the immune priming of invertebrates.

The Chinese mitten crab *Eriocheir sinensis* is a vital aquaculture species of critically evolutional significance and economic importance. The frequent outbreak of disease resulting from infection by the gram-negative pathogen *Aeromonas hydrophila* has caused drastic mortality and catastrophic economic losses in crab aquacultures. Like other invertebrates, crabs depend on only innate immunity to defend against invading pathogens, which includes a cellular response mediated by haemocytes and a humoral immune response that employs constitutive and inducible antimicrobial peptides (AMPs) to lyse invading microorganisms (15). As crabs lack adaptive immune response and vaccination protection against *E. sinensis*, knowledge about the persistence and mechanism of immune priming is urgently needed for the development of disease management strategies. The immune priming phenomenon in crab *E. sinensis* has been previously reported, and an increased survival rate has been associated with the significantly elevated phagocytosis of haemocytes as well as increased antibacterial activity in the cell-free haemolymph (16). However, the underpinning molecular mechanism of immune priming in crab is still not well understood. The circulating cell-free haemolymph plays a key role in the systemic immune protection of host, and increased specific antibodies in haemolymph are indispensable for immune protection in vertebrates after a pre-espouse. In invertebrates, especially in crabs, the candidate molecules responsible for the humoral protection during immune priming are still not understood. To establish which factors are responsible for these protective effects in primed haemolymphs, the present study undertook a proteomic analysis of cell-free haemolymphs before and after priming stimulation in crabs to identify candidate molecules. The key molecules in haemolymph and possible regulatory pathways involved in immune priming were investigated with the objective of providing insights into the mechanisms of immune memory in invertebrates, and strategies for disease prevention in the Chinese mitten crab *E. sinensis*.

## Materials and Methods

### Animals Culture and Bacteria Prepare

Chinese mitten crabs, *E. sinensis*, averaging about 30 g in wet weight, were collected from a local farm in Lianyungang, Jiangsu province, China, and maintained in aerated fresh water for one week before processing. Gram-negative bacteria *A. hydrophila* were incubated in LB medium at 28°C to logarithmic growth phases and harvested by centrifugation at 5000 × *g*, 4°C for 5 min. The cell pellet was washed three times with sterilized 0.85% normal saline and re-suspended in the same saline at a concentration of 6×10^7^ CFU mL^-1^ for the following experiments.

### Priming Stimulation of Crabs and Haemolymph Transfer

**Figure 1:**
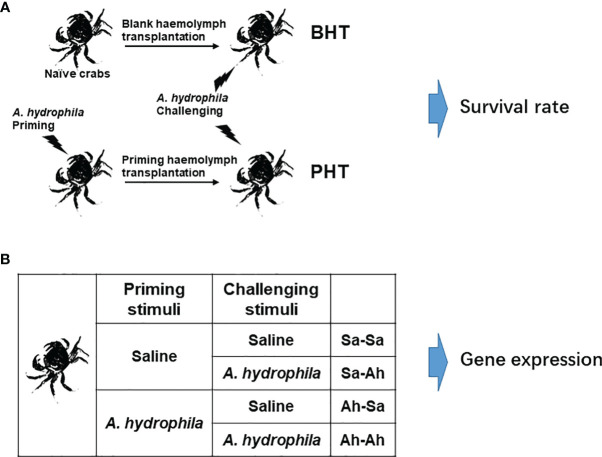
Experimental design. **(A)** *A. hydrophila* priming stimulation and haemolymph transfer. The crabs were primed by individually injecting with *A. hydrophila* and the haemolymph was sampled at 7 days (Ah-7d) post injection. Then cell-free haemolymph collected from Ah-7d crabs (priming haemolymph) and blank crabs (blank haemolymph) was transferred to the recipient, and seven days later the crabs were challenged by *A. hydrophila* injection. Then the survival rate of crabs (N=25) in each group was statistically recorded to 84 h. **(B)** The twice stimulation experiment in crab *E. sinensis*. The crabs were separated into 4 groups. The crabs in the Ah-Sa and Ah-Ah groups were first primed by 100 μL low dose *A. hydrophila* (6×10^6^ CFU mL^-1^) stimulation, while the crabs in the Sa-Ah and Sa-Sa groups received injections with saline as control. Then, 7 days later, the crabs in the Ah-Ah and Sa-Ah groups received *A. hydrophila* (6×10^7^ CFU mL^-1^) challenging stimulation, while the crabs in the Ah-Sa and Sa-Sa groups received injection with saline as control. At 12 h post the second injection, the haemolymph of crabs from the four groups (Sa-Ah, Ah-Ah, Sa-Sa, Ah-Sa) was collected for analysis. (Sa, saline; Ah, *A. hydrophila*; the first two letters mean priming regent, while the two latter letters mean challenging regent).

The crabs were primed with *A. hydrophila* and their cell-free haemolymph was transferred into the blank ones to examine the humoral protective effect. The suspension of *A. hydrophila* was diluted by 10 times with saline solution (~6×10^6^ CFU mL^-1^) which was used for priming stimulation. Nine crabs that received individual injections with 100 μL diluted *A. hydrophila* suspension were employed as the *A. hydrophila* priming group, and the nine crabs without any treatment were deemed the blank group. These crabs were maintained in aerated fresh water and sampled at day 7 post priming stimulation. The haemolymph from nine individuals in each group was sampled with an equal volume of pre-cold anticoagulant (27 mmol L^-1^ sodium citrate, 336 mmol L^-1^ NaCl, 115 mmol L^-1^ glucose, 9 mmol L^-1^ EDTA, pH 7.0) (**Figure 1A**), and pooled together as one sample. The haemolymph was centrifuged at 800 × *g*, 4°C for 10 min to separate the haemocytes and cell-free haemolymph. The cell-free haemolymph was stored immediately at -80°C for the following haemolymph transfer.

The haemolymph transfer experiment was conducted according to a previous report to investigate the enhanced humoral immune protection in crabs (17, 18). The total protein content of the cell-free haemolymph collected from priming group and blank group were quantified by BCA Protein Assay Kit (P0012S, Beyotime, China) and adjusted to the concentration of 5 mg mL^-1^ with saline solution, which were used as priming haemolymph and blank haemolymph, respectively. The untreated crabs were employed as the Naive crab (Naive) group. Two hundred microliters of priming haemolymph and blank haemolymph were injected into 25 recipient crabs in the priming haemolymph transferred (PHT) group and blank haemolymph transferred (BHT) group, respectively (**Figure 1A**). After acclimation for 24 hours, each of the 25 crabs in the three groups (Naïve, PHT, and BHT crabs) received an injection of 100 μL live *A. hydrophila* suspension (~6×10^7^ CFU mL^-1^). The survival rate of crabs (N=25) in each group was statistically recorded every 6 h until 84 h after the injection of *A. hydrophila*.

### Proteomic Analysis of Cell-Free Haemolymph From Crab

The proteomic analysis of haemolymph were conducted to find the possible candidate proteins responsible for the enhanced humoral protection. These candidate proteins were suspected to respond to immune stimulation by *A. hydrophila* and even keep a higher expression level over the physiological level when recovered from the response. The components of haemolymphs from crabs in immune defense and recovery were analyzed with priority. Eighteen crabs were priming stimulated with an injection of 100 μL diluted *A. hydrophila* suspension. Then nine crabs were randomly collected at 12 hours (time in immune defense) and 7 days (time recovered) post priming stimulation, respectively, which was deemed as Ah-12h group and Ah-7d group. Nine crabs without any treatment were deemed as the blank group. The haemolymph from three individuals in each of the three groups was sampled and pooled together (as one replicates) as the description of 2.2. A total of three replicates with nine individuals in each group were prepared for the following iTRAQ and LC-ESI-MS/MS proteomics analysis according to a previous report (19). iTRAQ data from three biological replicates were analyzed by MASCOT 2.3.02 software, and the protein identification was performed using the most recently updated *E. sinensis* SwissProt database. To reduce the probability of false peptide identification, only peptides with significance scores (≥20) at the 99% confidence interval by a Mascot probability analysis greater than “identity” were counted as identified peptides. For protein quantization, a protein must have contained at least two unique peptides. The quantitative protein ratios were weighted and normalized by the median ratio in Mascot. The ratios with a *p*-value of less than 0.05 were sorted, and examples with changes greater than 1.2 fold were considered significant. Functional annotations of the proteins were conducted using the Blast2GO program against the non-redundant protein database (NR; NCBI).

### Gene Cloning, Recombinant Expression and Antibody Preparing of *Es*ALFs

Sequence information of crab anti-lipopolysaccharide factor *Es*ALF-1 (GenBank accession gi|110671838) and *Es*ALF-3 (GenBank accession gi|325960711) was obtained from the national center for biotechnology information (http://www.ncbi.nlm.nih.gov/). The open reading frames (ORF) of *Es*ALF-1 and *Es*ALF-3 were amplified with the pairs of gene specific primers (**Table 1**). The PCR products were cloned into a pMD19-T vector (Takara, Japan) and sequenced in both directions with primers M13-47 and -RV (**Table 1**). The sequencing results were verified and subjected to cluster analysis. The cDNA sequence and deduced amino acid sequences of *Es*ALF-1 and *Es*ALF-3 were analyzed using the BLAST algorithm (http://www.ncbi.nlm.nih.gov/blast) and expert protein analysis system (http://www.expasy.org), respectively.

**Table 1:** Primers used for gene cloning, recombinant expression, RNAi, and Real-time PCR in this study.

Gene ID	Primer Sequence(5´-3´)
*Es*ALF-1 (gi:110671838)	Forward: TCAGGGGGAGACACAGACACAC
	Reverse: ACACATCAGGTCAGGATAGCCA
*Es*ALF-3 (gi:325960711)	Forward: ATTACCGTCTTCCAGCTTAGCG
	Reverse: ATTACCGTCTTCCAGCTTAGCG
*Es*ALF-1-*Eco*R V	Forward: CGGATATCATGAAGGACACATCAGGTCA
*Es*ALF-1-*Hin*d III	Reverse: CCAAGCTTAACCACCAGTATGGCACGCC
*Es*ALF-3-*Eco*R V	Forward: CGGATATCATGTTCAGCAAGGAAAGAAA
*Es*ALF-*3*-*Hin*d III	Reverse: CCAAGCTTGATCTTGAGCCAGAGTTTTG
*Es*ALF-1-RT (gi:110671838)	Forward: GGGTTAGCATCCTCCTGCGTCA
	Reverse: TGGCTGTTGCCGTGTTTACTCC
*Es*ALF-3-RT (gi:325960711)	Forward: TACCTCCCCCAGCCGTGTGA
	Reverse: TCTTGAGCCAGAGTTTTGCGTCC
*Es*Myd88-RT (AIM45535.1)	Forward: GAACAGGATGCCATTGGTGAAA
	Reverse: GGTGATCTTGAGGTGGATGTAGAGT
*Es*Pelle-RT (KP795393)	Forward: TAAGCCAGCAAACAACGGAGCA
	Reverse: GAGTCACAGGCAAAGAAGGGGA
*Es*TLR1-RT (JX295852)	Forward: CCACTGTCTTGCTCGTCGTCTT
	Reverse: CAATGCTCTGGTCAATCTGGTTCTG
*Es*TLR2-RT (KC011816)	Forward: GCATACCAGGACGACGAACAAG
	Reverse: TCAAGGAGGTCACAGTCACAGT
*Es*TLR1 (JX295852)	Forward: GAAGCAAGAGAAGCAGGGAGG
	Reverse: TCGTGGGGTCTGATGGCG
*Es*TLR2 (KC011816)	Forward: GCCCTCTTCCCACAGTTTCA
	Reverse: AGTCAAGGTAGATGAGAGAAGGGA
*Es*TLR1-RNAi (JX295852)	Forward: TAATACGACTCACTATAGGGACACACTTGGGGAGATTAGCC
	Reverse: TAATACGACTCACTATAGGGGCTTCTCTTGCTTCTGGCTTAC
EGFP-RNAi	Forward: GCGTAATACGACTCACTATAGGGCTGGACGGCGACG
EGFP-RNAi	Reverse: GCGTAATACGACTCACTATAGGTCAGGGCGGACTGGGTGCT
Actin	Forward: GCATCCACGAGACCACTTACA
Actin	Reverse: CTCCTGCTTGCTGATCCACATC

The recombinant expression and purification of proteins *Es*ALF-1 and *Es*ALF-3 were conducted as previous report (18). The complete cDNA fragments encoding the polypeptide of *Es*ALF-1 and *Es*ALF-3 were amplified and inserted into the expression vector pET-32a by T4 ligase (NEB, USA). The recombinant plasmids were extracted and then transfected into *Escherichia coli* Transetta (DE3) (TransGen Biotech). After screening, the positive transformants were incubated in LB medium containing ampicillin (100 μg mL ^-1^) at 37°C by shaking at 120 rpm for about seven hours. When the culture medium reached OD_600_ = 0.6, isopropyl β-D-1-Thiogalactopyranoside (IPTG) was added into LB medium at a final concentration of 1 mM and incubated at 37°C with shaking at 120 rpm for eight hours. The bacteria were harvested and the recombinant proteins of *Es*ALF-1 and *Es*ALF-3 (r*Es*ALF-1 and r*Es*ALF-3) were purified by affinity chromatography using His-resin as the previous report. The purified protein was separated by 12% SDS-poly-acrylamide gel electrophoresis (SDS-PAGE), and visualized with Coo-massie bright blue R250. The concentration of purified r*Es*ALF-1 and r*Es*ALF-3 were quantified by the BCA method. The purified r*Es*ALF-1 and r*Es*ALF-3 were dialyzed in PBS and then stored at -80°C for the preparation of polyclonal antibodies.

To acquire the polyclonal antibody, six-week-old female mice were immunized with 100 μg r*Es*ALF-1 and r*Es*ALF-3 according to the previous description (20). The r*Es*ALF-1, r*Es*ALF-3, and the cell-free haemolymphs sample from *E. sinensis* were used to determine the specificity of anti-*Es*ALF-1 and anti-*Es*ALF-1 antibodies by western blot.

### Successive Immune Stimulation of Crab *E. sinensis* by *A. hydrophila*

To explore the enhanced protective response upon re-infection and the biphasic immune response of immune related genes, two successive immune stimulations were conducted in crab *E. sinensis* according to a previous report (16). The first priming stimulation was conducted, with 18 crabs divided equally into two groups, which were individually treated by injection with 100 μL diluted suspension of *A. hydrophila* (~6×10^6^ CFU mL^-1^) or 100 μL saline solution. The crabs were maintained in aerated fresh water for seven days, and then individually received the second injection of 100 μL live *A. hydrophila* suspension (~6×10^7^ CFU mL^-1^) or 100 μL saline solution, respectively (**Figure 1B**). The crabs that received the twice saline solution injection or *A. hydrophila* injection were deemed the Sa-Sa group or Ah-Ah group, respectively. The crabs received the first saline solution injection and the second *A. hydrophila* injection was deemed as the Sa-Ah group. The crabs received the first *A. hydrophila* injection and the second saline solution injection was deemed the Ah-Sa group. Then haemolymph was collected from the four groups (Sa-Ah, Ah-Ah, Sa-Sa, Ah-Sa) with pre-cold anticoagulant at 12 h post the second injection as above description of 2.2. The haemocytes and cell-free haemolymph were separated by centrifugation as mentioned above. The haemocytes were stored in 1 mL Trizol regent at -80°C for the following detection of mRNA expression. The haemolymph was stored directly at -80°C for western-blotting analysis.

### Western Blotting Analysis of *Es*ALFs in Haemolymph

The cell-free haemolymph from crabs of Sa-Ah, Ah-Ah, Sa-Sa, and Ah-Sa group was analyzed by western blot to determine the protein expression levels of *Es*ALF-1 and *Es*ALF-3. Briefly, protein samples were separated by SDS-PAGE and electrophoretically transferred onto a 0.45 mm pore nitrocellulose membrane at 200 mA for 5 h. The membrane was blocked with PBS containing 3% BSA at 37°C for 1h and incubated with the prepared antibody at 37°C for 1 h. After being washed three times with PBS containing 0.05% Tween-20 (PBS-T), the membrane was incubated with goat anti-mice Ig-HRP conjugate (Southern Biotech, diluted1:5000 in PBS) at 37°C for 1 h. After the final three washes with PBS-T, the membrane was stained with Western Lightning ECL Pro (PerkinElmer). The intensity of the target protein was obtained by scanning the grey level of the protein band using Photoshop software and then adjusted by eliminating background values. Each experiment was conducted in triplicate.

### *In Vitro* and *In Vivo* Antibacterial Activity of *Es*ALFs

The *in vitro* assay was conducted based on the previous description to determine the antibacterial activity of *Es*ALFs (21). Briefly, 50 μL of bacteria resuspension was incubated with 50 μL r*Es*ALF-1, r*Es*ALF-3 supernatant (0.3 mg mL^-1^) and equal volume of control Tag-protein rTRX (0.3 mg mL^-1^) with shaking at room temperature for 30 min, respectively. Subsequently, 20 μL of the cultures were pipetted into a sterile 96 well plate with 200 μL of LB. The plate was incubated on a plate reader at 28°C for 12 h. The absorbance at 600 nm (OD_600_) was measured every half an hour, which was employed to indicate the growth of bacteria. The bacteria incubated with sterile PBS solution were used as a control. Each experiment was conducted in triplicate.

The *in vivo* antibacterial activities of *Es*ALFs was examined by injecting them into the crabs challenged by *A. hydrophila.* Fifty microliter of r*Es*ALF-1 (0.1 mg mL^-1^), r*Es*ALF-3 (0.1 mg mL^-1^) and control Tag-protein rTRX (0.1 mg mL^-1^) was injected into twenty-five crabs of three groups (r*Es*ALF-1, r*Es*ALF-3 and rTRX group), respectively. These crabs were challenged by an injection of 100 μL live *A. hydrophila* suspension (~6×10^7^ CFU mL^-1^) at 24 h post injection of proteins, and the survival rate of crabs was statistically recorded (N=25) every 6 hours. To further deplete the *in vivo* function of *Es*ALF-1 and *Es*ALF-3, 75 crabs were first primed by the injection of 100 μL live *A. hydrophila* suspension (~6×10^6^ CFU mL^-1^) to induce the expressions of *Es*ALFs as described previously and then divided into three groups (anti-*Es*ALF-1, anti-*Es*ALF-3, and pre-antibody group). After 7 days, the crabs in the three groups received an injection of 50 μL prepared anti-*Es*ALF-1 antibody (0.1 mg mL^-1^), anti-*Es*ALF-3 antibody (0.1 mg mL^-1^) and pre-antibody (0.1 mg mL^-1^) to deplete *Es*ALFs, respectively. At 24 hours after the injection of antibodies, the crabs were challenged with 100 μL live *A. hydrophila* (~6×10^7^ CFU mL^-1^) as mentioned above, and the survival rate of crabs was statistically recorded (N=25) every 6 hours.

### RNA Interfere to Knock Down Gene Expression

To validate whether the expression of *Es*ALF-1 and *Es*ALF-3 was regulated by Toll-like receptor (TLR) signal during the immune response of crabs, double strand RNA interference (dsRNAi) strategy was adopted to disrupt the mRNA expression of *Es*TLR1 according to a previous report (21). The DNA fragments of *Es*TLR1 and enhanced green fluorescent protein (EGFP, 657 bp) were amplified with T7 promoter linked primers (**Table 1**) from crab genomic DNA and pEGFP vector, respectively. The dsRNAs of *Es*TLR1 and EGFP were synthesized using *in vitro* transcription T7 kit (Takara, Japan) following the instruction. Finally, the dsRNA was dissolved in PBS at a final concentration of 1 μg μL^-1^. Crabs were randomly separated into three groups (PBS, dsEGFP, and dsTLR1 groups), which received the injection of 100 μL PBS, EGFP-dsRNA, or *Es*TLR1-dsRNA, respectively. At 24 h post injection, haemocytes were collected to determine the mRNA expressions of *Es*TLR1 and its downstream *Es*Myd-88, *Es*Pelle, *Es*ALF-1, and *Es*ALF-3.

### The Lasting Up-Regulation of Immune Genes After Priming Stimulation

To detect the lasting protection effects of priming stimulation, the expressions of immune genes in haemocytes were monitored at different times (one to four weeks later). Thirty-six crabs were priming stimulated with the injection of 100 μL diluted *A. hydrophila* suspension. Then nine crabs were randomly collected at 7 days, 14 days, 21 days, and 28 days post priming stimulation, respectively, and deemed the Ah-7d group, Ah-14d group, Ah-21d group, and Ah-28d group. Nine crabs without any treatment were deemed the blank group. The haemolymph from three individuals in each of the three groups was sampled and pooled together (as one replicate) as described previously. There were three replicates for each group. The haemolymph was centrifuged at 800 × *g*, 4°C for 10 min to separate the haemocytes and cell-free haemolymph. The haemocytes were stored immediately in 1 mL Trizol regent -80°C for subsequent detection of gene expression.

### Quantitative Real-Time PCR

Total RNA was extracted from muscle, hepatopancreas, haemopoietic tissue, gill, and haemocytes and reverse transcribed into cDNA using the Prime-Script™ real-time PCR kit (Takara, Japan) according to the instructions. The spatial distribution of *Es*ALF-1 and *Es*ALF-3 mRNA in the five tissues, and the mRNA expressions of *Es*ALF-1, *Es*ALF-3, *Es*My88, *Es*Pelle, and *Es*TLR1 in haemocytes after immune priming or knockdown of *Es*TLR1 expression were determined by qRT-PCR with specific primers (**Table 1**), respectively. The qRT-PCR reactions were conducted with an ABI PRISM 7500 Sequence Detection System (Applied Biosystems) using SYBR green fluorescent (Takara, Japan) according to the instruction. A 267 bp fragment of *Es*β-actin (GenBank accession No. HM053699) amplified with the specific primers, *Es*β-actin-RT-F and -R (**Table 1**) was employed as endogenous control. The qRT-PCR reactions operated as follows: 95°C for 10 min, followed by 40 cycles at 95°C for 10 s and 60°C for 45 s. To confirm the specificity of PCR products, the dissociation curve analysis of amplification products was performed at the end of each PCR. The relative mRNA expression level was analyzed by comparative Ct method (2 ^-△△Ct^ method) (22).

### Statistical Analysis

The survival rate was calculated with a Kaplan-Meier estimate followed by a log-rank test in SPSS 17.0. All data were subjected to a one-way analysis of variance (one-way ANOVA) followed by a *post hoc* multiple comparisons (Tukey’s) test. Differences were considered significant at *p* < 0.05.

## Results

### The Increased Survival Rate of Recipient Crabs After Priming Haemolymph Transfer

**Figure 2:**
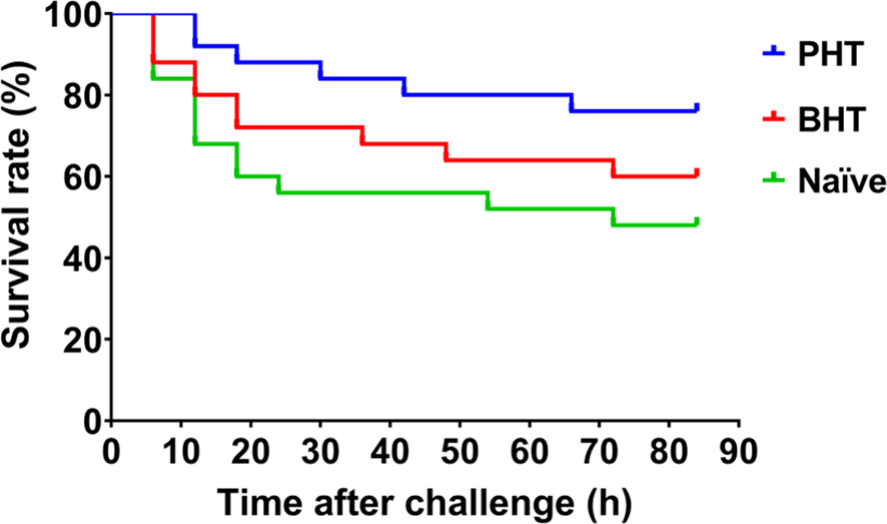
The survival rate of recipient crabs after the transfer of primed haemolymph improved. Compared with naïve crabs (Naive) or blank haemolymph transferred (BHT) crabs, the survival rate of Primed haemolymph (PHT) recipient crabs was increased after *A. hydrophila* challenge.

The survival rates of crabs in the priming haemolymph transferred group (PHT), blank haemolymph transferred group (BHT), and naïve crab group (Naïve) were monitored after the crabs were challenged with live bacteria *A. hydrophila*. The mortality of crab was observed 6 h post challenge in the Naïve and BHT groups, and later at 12 h in the PHT group. A total of 13 individuals died in the Naïve group, which was more than that in the BHT group (10 individuals) and the PHT group (6 individuals). The log-rank test showed a significant difference in survival rate between the PHT group and the other two groups after live *A. hydrophila* challenge (*p* < 0.05, Chi square = 4.640, df = 1), (**Figure 2**). No more additional mortality was observed in all the groups after 72 h post challenge and no crabs died in the untreated crabs (data not shown).

### Anti-lipopolysaccharide Factors (ALFs) Identified in the Primed Haemolymph by Proteomic Analysis

**Figure 3:**
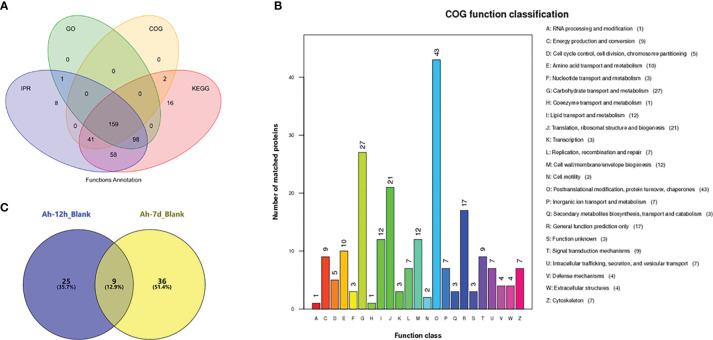
Haemolymph proteins identification by iTRAQ proteomic analysis. **(A)** Functional annotation of identified haemolymph proteins based on the four databases, in which 383 proteins were functionally annotated. **(B)** The haemolymph proteins could be functionally classified into 23 classes based on COG annotation. **(C)** Quantitative expression analysis of haemolymph proteins, and there were 34 and 45 differentially expressed proteins in haemolymph of crabs at 12 hours (Ah-12h) and 7 days (Ah-7d) post priming stimulation, compared with that in blank crabs, respectively.

iTRAQ proteomic analyses of cell-free haemolymph from blank crabs (blank group) and crabs at 12 h (Ah-12h group) and 7 days (Ah-7d group) post priming stimulation were conducted to establish a complete quantitative and qualitative protein expression database for crab haemolymph. A total of 474 proteins were identified in the haemolymph of crab, and 383 of them could be functionally annotated in the four online database (**Figure 3A**). Based on COG database annotation, the haemolymph proteins could be functionally classified into 23 classes, and most of them were related to chaperones, transportation, metabolism, and defense, such as transport and metabolism of carbohydrate, amino acid, lipid, nucleotide, and inorganic ion, signal transduction mechanisms and defense mechanisms (**Figure 3B**). Quantitative expression analysis of these proteins revealed that, compared with control crabs, a total of 70 proteins were differentially expressed in haemolymph after priming stimulation, with 34 and 45 ones at 12 h (Ah-12h) and 7 days (Ah-7d) post priming stimulation, respectively (**Figure 3C**) (**Table 2**). And nine of them, including anti-lipopolysaccharide factor 3 (ALF3), Apolipophorin and cystatin, were all differentially expressed in the Ah-12h group and Ah-7d group when compared with that in blank group.

**Table 2:** Number of differentially expressed proteins (DEPs) between different groups.

Group	Num. of Total Quant	Num. of Total Sig.	Num. of Sig. Up	Num. of Sig. down
Ah-12h	372	34	13	21
Ah-7d	214	45	27	18

**Figure 4:**
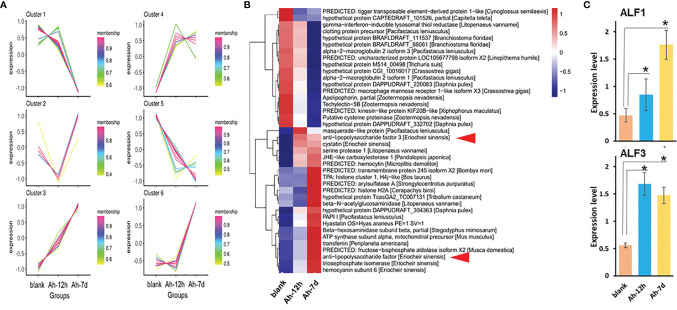
Anti-lipopolysaccharide factors (ALFs) identified in primed haemolymph by proteomic analysis. The cell-free haemolymph collected from blank (Blank) crabs or crabs received *A. hydrophila* priming stimulation at 12 hours (Ah-12h) and 7 days (Ah-7d) was analyzed by proteomics. A total of 70 differentially expressed proteins (DEPs) were identified with many metabolic or immune related components annotated. Based on their expression changes after *A. hydrophila* priming stimulation, there were six expression modules **(A)** of these DEPs, and the expression heatmap showed more than half of up-regulated DEPs **(B)**. Among them, two ALFs (*Es*ALF-1 and *Es*ALF-3) were significantly up-regulated **(C)**. Vertical bars represent the mean ± S.D. (N = 3). *p < 0.05.

Based on the expression levels in haemolymph, about half of these 70 proteins were up-regulated after priming stimulation, and there were six modules of expression pattern revealed (**Figure 4A**). The proteins in cluster 3 and cluster 4 were all up-regulated in the Ah-12h group and Ah-7d group compared with the blank group. Except for 27 unknown proteins, a number of immune related proteins were annotated, such as two alpha-2-macroglobulin, hyastatin, serine protease 1, macrophage mannose receptor, and histone H2A (**Figure 4B**). Among the up-regulated proteins, two anti-lipopolysaccharide factors (ALF1 and ALF3) were sorted by highest expression (**Figure 4B**). Based on the proteomic data, the expressions of ALF1 and ALF3 in haemolymph from Ah-12h and Ah-7d crabs were significantly (*p* < 0.05) increased by 1.80- and 3.74-fold, and 3.00- and 2.63-fold compared to the control crabs, respectively (**Figure 4C**).

### The Expressions of *Es*ALFs in Haemocytes and Its Significant Increase After Priming Stimulation

**Figure 5:**
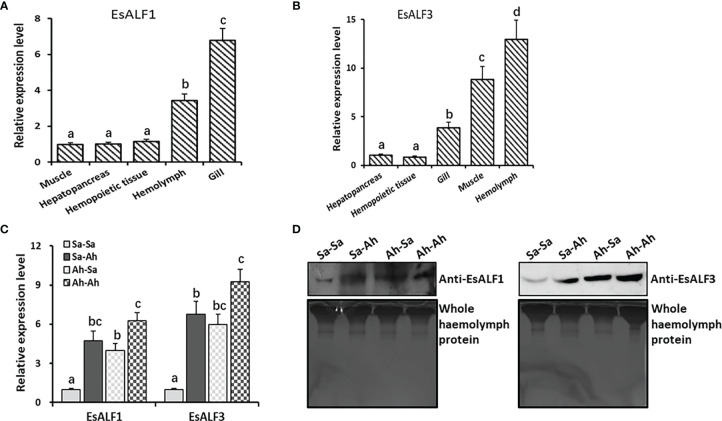
High expression of ALFs in haemocytes and significant induction after priming stimulation. **(A)** The expression pattern of *Es*ALF-1 in different tissues of crab *E. sinensis*; **(B)** The expression pattern of *Es*ALF-3 in different tissues of crab *E. sinensis*; **(C)** The mRNA expression levels of *Es*ALF-1 and *Es*ALF-3 in haemocytes after stimulation of *A. hydrophila*; **(D)** WB analysis of the protein expression levels of *Es*ALF-1 and *Es*ALF-3 in cell-free haemolymph after stimulation of *A. hydrophila*, with whole haemolymph protein used as the internal reference. Vertical bars represent the mean ± S. D. (N = 3), the letters (a, b, c, d) were used to present significant differences (p < 0.05).

The coding sequences of the *Es*ALF-1 and *Es*ALF-3 were cloned, recombined, and expressed in *E. coli* Transetta (DE3). The recombinant proteins (r*Es*ALF-1 and r*Es*ALF-3) were used to prepare specific polyclonal antibodies. The mRNA expression levels of *Es*ALF-1 and *Es*ALF-3 in different tissues of crabs were examined by RT-PCR. *Es*ALF-1 was highly expressed in gill and haemocytes, while *Es*ALF-3 was mainly expressed in haemocytes (**Figures 5A, B**). Their mRNA expressions in haemocytes and protein contents in cell-free haemolymph were also detected after successive stimulation of *A. hydrophila*. The mRNA transcripts of *Es*ALF-1 and *Es*ALF-3 were significantly (*p* < 0.05) induced in both the Sa-Ah group (4.74 and 6.72-fold of those in Sa-Sa) and Ah-Ah group (6.23 and 9.27-fold of those in control group Sa-Sa) after *A. hydrophila* challenge, compared with those in the control group (Sa-Sa) (**Figure 5C**). While in the *A. hydrophila* primed group (Ah-Ah), the mRNA expression level of *Es*ALF-3 (9.27 times of that in Sa-Sa), but not *Es*ALF-1, was significantly (*p* < 0.05) higher than that (6.72–fold of that in Sa-Sa) in the un-primed group (Sa-Ah) (**Figure 5C**). After *A. hydrophila* priming, the expression of *Es*ALF-1 (3.89-fold of that in Sa-Sa) and *Es*ALF-3 (5.99 times of that in Sa-Sa) were both significantly (*p* < 0.05) up-regulated 7 days post stimulation, even without *A. hydrophila* challenge (**Figure 5C**). The abundance of *Es*ALF-1 and *Es*ALF-3 proteins in cell-free haemolymph also increased after *A. hydrophila* challenge in the primed (Ah-Ah) and un-primed (Sa-Ah) groups compared with those in the control (Sa-Sa) group (**Figure 5D**). In *A. hydrophila* primed groups (Ah-Sa and Ah-Ah), much higher expression levels of *Es*ALF-3 were detected, compared to that in the un-primed groups (Sa-Sa and Sa-Ah) (**Figure 5D**).

### The *In Vitro* and *In Vivo* Antibacterial Activities of *Es*ALFs

**Figure 6:**
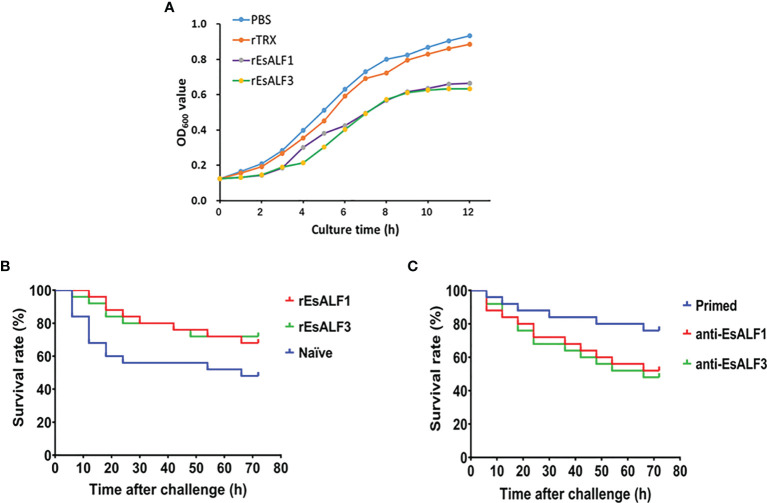
The antibacterial activities of ALFs and impaired resistance after ALFs depletion in haemolymph. **(A)** The anti-bacterial activities of r*Es*ALF-1 and r*Es*ALF-3 toward *A. hydrophila*; **(B)** The survival rate of blank crabs received r*Es*ALF-1 or r*Es*ALF-3 injection (*in vivo* over-expression of ALFs); **(C)** The survival rate of primed crabs (crabs at 7 days after *A. hydrophila* priming) received anti-*Es*ALF-1 or anti-*Es*ALF-3 antibody injection (*in vivo* depletion of ALFs).

The anti-bacterial activities of r*Es*ALF-1 and r*Es*ALF-3 were determined by incubation with bacteria *A. hydrophila*. The growth of *A. hydrophila* was significantly inhibited after it was pre-incubated with r*Es*ALF-1 or r*Es*ALF-3 protein (**Figure 6A**). The *in vivo* anti-bacterial activities of r*Es*ALF-1 and r*Es*ALF-3 were examined by directly injecting them into the haemocoele of crabs, which were challenged by *A. hydrophila* 24 h after protein injection. The survival rates of r*Es*ALF-1 and r*Es*ALF-3-injected crabs were 68% and 72%, which were significantly (Chi square = 3.640, df = 1, and *P* value = 0.0432) higher than that (48%) in the rTRX injected control group (**Figure 6B**). To further deplete the *in vivo* function of *Es*ALF-1 or *Es*ALF-3 in primed crabs (Ah-7d), the anti-r*Es*ALF-1 antibody and anti-r*Es*ALF-3 antibody were injected into the haemocoele of crabs, respectively. Compared with pre-antibody injected control group (76%), the survival rate of primed crabs in *Es*ALF-1 or *Es*ALF-3 depleted group was significantly (Chi square = 3.896, df = 1, and *P* value = 0.0484) reduced, which was about 52% and 48% respectively (**Figure 6C**).

### The Expression of *Es*ALFs After Priming Stimulation and Knockdown of *Es*TLR1 Expression

**Figure 7:**
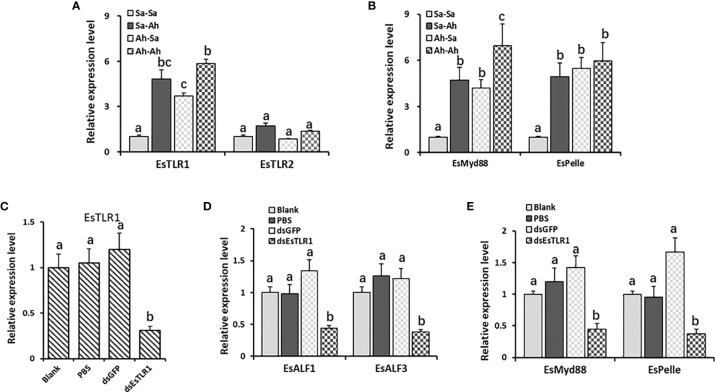
The key roles of TLR1 signal in regulating the expressions of ALFs. **(A)** The expression levels of *Es*TLR1 and *Es*TLR2 after *A. hydrophila* priming and challenging stimulation; **(B)** The expression levels of TLR signal component *Es*Myd88 and *Es*Pelle after *A. hydrophila* priming and challenging stimulation; **(C)** The expression levels of *Es*TLR1 after RNAi by ds*Es*TLR1 injection; **(D)** The expression levels of *Es*ALF-1 and *Es*ALF-3 after *Es*TLR1 inhibition; **(E)** The expression levels of TLR signal component *Es*Myd88 and *Es*Pelle after *Es*TLR1 inhibition. Vertical bars represent the mean ± S. D. (N = 3), the letters (a, b, c) were used to present significant differences (p < 0.05).

The expressions of the key components of the TLR signaling pathway (including *Es*TLR1, *Es*TLR2, *Es*Myd88, and *Es*Pelle) in the haemocytes of oysters after priming stimulation and RNA interference of *Es*TLR1 were detected by RT-PCR to find the possible regulation pattern of *Es*ALF-1 and *Es*ALF-3 expressions. The expression levels of *Es*TLR1, *Es*Myd88, *Es*Pelle but not *Es*TLR2, were significantly (*p* < 0.05) increased in Sa-Ah and Ah-Ah group after *A. hydrophila* challenge, compared with that in Sa-Sa group (**Figures 7A, B**). Moreover, the expressions of *Es*Myd88 and *Es*ALF-3 in *A. hydrophila* primed crabs (Ah-Ah group) were even much higher (*p* < 0.05) than those of the un-primed Sa-Ah group after challenging stimulation, while those of *Es*TLR1, *Es*Pelle, and *Es*ALF-1 were not. After priming, the expressions of *Es*TLR1, *Es*Myd88, and *Es*Pelle in the Ah-Sa group were still significantly higher at 7 days post priming stimulation than those in the Sa-Sa group, even without challenging stimulation (**Figures 7A, B**).

After the expression of *Es*TLR1 was knocked down by using RNAi strategy (**Figure 7C**), the expressions of *Es*My88, *Es*Pelle, *Es*ALF-1 and *Es*ALF-3 in the ds*Es*TLR1 group were all significantly (*p* < 0.05) inhibited (**Figures 7D, E**), which was 0.32, 0.22, 0.33, and 0.30-fold of that in the dsGFP group, respectively.

### The Lastingly Enhanced Expression of ALFs in Crab After Priming Stimulation

**Figure 8:**
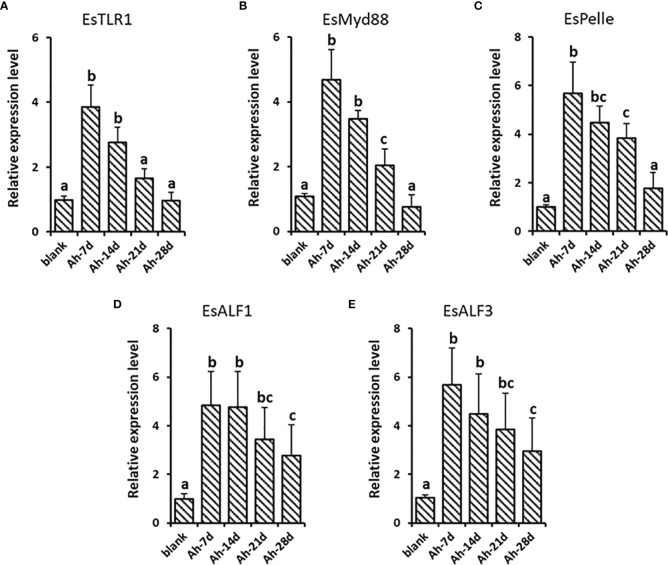
The lastingly enhanced expression of ALFs in crab after priming stimulation. After *A. hydrophila* priming stimulation, the lasting expressions of *Es*TLR1 **(A)**, *Es*Myd88 **(B)**, *Es*Pelle **(C)**, *Es*ALF-1 **(D)** and *Es*ALF-3 **(E)** were detected at day 7 (Ah-7d), day 14 (Ah-14d), day 21 (Ah-21d) and day 28 (Ah-28d). Vertical bars represent the mean ± S. D. (N = 3), the letters (a, b, c) were used to present significant differences (p < 0.05).

The gene expression levels of *Es*TLR1, *Es*My88, *Es*Pelle, *Es*ALF-1, and *Es*ALF-3 were monitored at 7, 14, 21, and 28 days post *A. hydrophila* priming stimulation to validate the lasting effect. The expressions of *Es*TLR1 and *Es*My88 were significantly (*p* < 0.05) increased at 7 and 14 days post *A. hydrophila* priming, while declined at 21 and 28 days, which was 3.84, 2.76, 1.64, 0.96 and 4.68, 3.48, 2.05, 0.76-fold of that in the blank group, respectively (**Figures 8A, B**). *Es*Pelle was also significantly (*p* < 0.05) highly expressed till 21 days post *A. hydrophila* priming, which was 3.84-fold of that in the blank group (**Figure 8C**). For *Es*ALF-1 and *Es*ALF-3, they kept significantly (*p* < 0.05) higher expression levels until 28 days post priming stimulation, which was 2.76 and 2.95-fold that in the blank group (**Figures 8D, E**).

## Discussion

Enhanced protection after a previous immune stimulation has so far been reported in various invertebrates, including Annelida, Nematoda, Mollusca, and Arthropoda (4, 5). However, the involved molecules and possible regulation mechanisms are still unclear. Though some candidate variable proteins such as DSCAM, FREP, and VCBP have been identified (23, 24), their functions cannot fully cover the features of immune memory, indicating that these molecules might not be the common mechanisms of immune priming in invertebrates (6). It is suspected that different molecules and strategies might be adopted by invertebrates, which is quite fascinating and crucial to understanding the evolution of immune memory. The increasing evidence of immune priming in invertebrates suggests that both the humoral and cellular immune responses could be responsible for enhanced immune protection. A preliminary study in our laboratory has revealed the enhanced humoral protection in crab *E. sinensis* (16), and there could be some factors responsible for the protective effects in primed haemolymph. The possible molecules and strategies involved in the enhanced protection are further investigated in the present study.

A cell-free haemolymph transfer experiment was first conducted to verify the protective effect of humoral immunity in crabs. The survival rate of naïve crabs receiving priming haemolymph transfer was significantly increased after *A. hydrophila* challenge, compared with that of blank haemolymph transferred crabs or naïve crabs, indicating the protective effect the cell-free haemolymph from primed crabs. Though the specificity of this protective effect in priming haemolymph was not detected in the present study, it could be confirmed at least that the immune enhancement of priming haemolymph was present in *A. hydrophila* primed crabs. As the priming haemolymph was collected at 7 days post the first *A. hydrophila* priming stimulation, the protective effect was confirmed to be persistent for at least 7 days. It has also been reported in the insect *Galleria mellonella* that, the antibacterial factors in the haemolymph of donor individuals could be transferred into recipients (18, 25). There could also be some immune factors in the priming haemolymph of crabs responsible for the protective effect. Moreover, the transfer of haemolymph from blank crabs could also, to some extent, provide immune protection and improve survival. A similar result was also reported in *G. mellonella* (18), which might be attributed to basal anti-bacterial activity in blank haemolymph. Some factors could be responsible for these protective effects in primed or blank haemolymph. Therefore, the proteomic analysis of cell-free haemolymph before and after priming stimulation was further conducted, to identify candidate molecules.

The haemolymph proteins are key components of the circulating system, which play important roles in nutrition transporting, metabolism, and maintaining homeostasis. By iTRAQ and LC-ESI-MS/MS proteomic analysis, there were 474 proteins identified from haemolymph of crab *E. sinensis*. By COG annotation, a number of proteins were identified in the haemolymph proteins, and most of them were associated with transport and metabolism, such as carbohydrate, amino acid, lipid, nucleotide, and inorganic ion transport and metabolism. The circulating cell-free haemolymph plays key roles in nutriment transporting, metabolism, and the systemic immune protection of the host. These molecules are identified as indispensable for the function of these processes. There were 34 (Ah-12h group) and 45 (Ah-7d group) differentially expressed proteins induced by priming stimulation, and most of them were annotated to be involved in circulating (hemocyanin and clotting protein precursor), metabolism (transferrin, beta-N-acetylglucosaminidase, beta-hexosaminidase, and JHE-like carboxylesterase) and immunity (hyastatin, anti-lipopolysaccharide factors, serine protease 1, histone H2A, alpha-2-macroglobulin, macrophage mannose receptor). These results indicate that, except for immune reactions, circulating and metabolism processes also participated and played fundamental roles in the immune priming of crabs. It has been reported that metabolism plays important regulatory roles during innate memory formation in vertebrates (24), and that metabolism-related genes were highly expressed in the haemocytes of primed oysters (12). Most of the immune-related proteins were found to be up-regulated after priming stimulation, even 7 days post stimulation. These results indicated that various proteins in haemolymph and immune reactions were involved in the protective effects of crab immune priming. Two anti-lipopolysaccharide factors (ALF1 and ALF3) were identified after screening the highly up-regulated immune molecules, and their involvement in the immune priming was further investigated.

The physical functions of *Es*ALF-1 and *Es*ALF-3 were further explored in crabs. First, the distribution of *Es*ALF-1 and *Es*ALF-3 mRNA in different tissues of blank crabs were determined, and they were both found to be highly expressed in immune related tissues, such as haemocytes, gill, and *Es*ALF-3 mRNA were even more concentrated in haemocytes. In the genome of crab *E. sinensis*, three genes are encoded and ALF was identified. They were differentially expressed in the tissues of the crab with various functions (26, 27). In the present study, after first priming and second challenging stimulation with *A. hydrophila*, the expressions of *Es*ALF-1 and *Es*ALF-3 were significantly induced at both mRNA and protein levels, indicating that *Es*ALF-1 and *Es*ALF-3 could promptly respond to the stimulation of *A. hydrophila*. In oyster *C. gigas*, the expressions of anti-bacterial effectors *Cg*TIMP and *Cg*PRTP were elevated after *V. splendidus* priming stimulation and suggested to participate in the immune priming process (12). In snail *B. glabrata*, the humoral biomphalysin was highly expressed after S. mansoni pre-treatment and was required for the development of innate memory (14). It could be inferred that varied molecules are needed for the formation of immune priming in different organisms. The expression levels of *Es*ALF-1 and *Es*ALF-3 in primed crabs (Ah-Sa) were still much higher than that in naïve crabs (Sa-Sa) on the seventh day after *A. hydrophila* priming without challenging stimulation. The expression of *Es*ALF2 may not change significantly during immune priming. These results were consistent with proteomic data, which confirmed stored expressions of *Es*ALF-1 and *Es*ALF-3 in both haemocytes (at mRNA level) and haemolymph (at protein level) after *A. hydrophila* priming. The stored expressions of *Es*ALF-1 and *Es*ALF-3 proteins in haemolymph might underpin the higher resistance of priming-haemolymph transferred crabs against *A. hydrophila* challenge. The expression levels of *Es*ALF-1 and *Es*ALF-3 in *A. hydrophila* primed crabs (Ah-Ah) were much higher than that in naïve crabs (Sa-Ah) after challenging stimulation. The results indicated that *Es*ALF-1 and *Es*ALF-3 might play important roles in the immune memory induced by *A. hydrophila* stimulation. *Es*ALF-1 and *Es*ALF-3 were previously reported to exhibit anti-bacterial activities (15, 27), and their functions were further validated in the present study with recombinant proteins of r*EsALF-1* and r*EsALF-3*, which displayed comparable inhibitory effects on the growth of *A. hydrophila*. r*Es*ALF-1 and r*Es*ALF-3. Anti-r*Es*ALF-1 and anti-r*Es*ALF-3 antibodies were further injected into the haemocoele of crabs to explore the *in vivo* function of *Es*ALF-1 or *Es*ALF-3, respectively. The over acquisition of *Es*ALF-1 or *Es*ALF-3 in naïve crabs could significantly improve their survival rate, while the depletion of *Es*ALF-1 or *Es*ALF-3 with corresponding antibodies in the primed crabs (Ah-7d) could significantly impair their resistance against *A. hydrophila* challenge. All the results indicated that *Es*ALF-1 and *Es*ALF-3 played key roles in the enhanced priming protection of crabs.

It has been reported that the TLR signal is involved in the immune priming of invertebrates. TLR signaling is a conserved and canonical innate immune activation pathway, which has been extensively studied in various organisms, and has been reported to control the expression of antimicrobial peptides induced by bacterial and fungal infections in invertebrates (28, 29). In *Drosophila*, the toll pathway but not the Imd pathway is required for primed response to *S. pneumonia* (9). In oyster *C. gigas*, TLR signal was found to be involved in enhanced immune protection (12). Two TLRs were identified in crab *E. sinensis* and they were reported to respond against the stimulation of lipopolysaccharide, peptidoglycan, and zymosan (30). In the present study, the expressions of the examined key components of the TLR signaling pathway (including *Es*TLR1, *Es*TLR2, *Es*Myd88, and *Es*Pelle) were all (except for *Es*TLR2) significantly up-regulated after the first priming and second challenging stimulation. It could be inferred that the *Es*TLR1 (but not the *Es*TLR2) signal participated in the enhanced protection induced by *A. hydrophila* in crab *E. sinensis*. *Es*ALF-3 and *Es*Myd88 were even significantly higher expressed in a similar challenge (Ah-Ah) than a different challenge (Sa-Ah), indicating a biphasic immune response and priming effect after *A. hydrophila* stimulation. While *Es*TLR1, *Es*Pelle, and *Es*ALF-1 were not significantly induced in second stimulation than in first, which might not play core roles in this process. TLR signal has been reported to regulate the expression of AMPs for anti-bacterial immunity in *Drosophila* (31). To confirm the regulation of the *Es*TLR1 signal on the production of *Es*ALF-1 and *Es*ALF-3 during priming response, the mRNA expression of *Es*ALF-1 and *Es*ALF-3 were examined after *Es*TLR1 was knocked down by ds*Es*TLR1 injection. In *Es*TLR1-knockdown crabs, the expressions of *Es*My88, *Es*Pelle, and the two ALFs (*Es*ALF-1 and *Es*ALF-3) were all inhibited. These results collectively indicate that the enhanced expressions of *Es*ALF-3 in immune priming of crabs were regulated by the higher expressed *Es*TLR1 signal, and *Es*TLR1-mediated production of *Es*ALF-3 was the key step for *A. hydrophila* priming induced protection in crabs.

The long-lasting even life-long protection of vaccines has been widely reported in vertebrates. However, the persistence of this enhanced immune protection induced by pre-exposure in invertebrates it is still not clear. In shrimp *Litopenaeus vannamei*, the vaccination effects of pre-exposure to *V. alginolyticus* could provide protection for primed individuals for as long as four weeks (32). The short-lasting effect of immune priming was also reported in *Drosophila*, and the duration of immune protection was priming manner dependent (33). The protective immune response could last for more than ten days after exposure to a persistent low-in-virulence live pathogen infection, but it was eliminated seven days after the host was primed with heat-killed bacteria (32). In the present study, the expression changes of the *Es*TLR1 signal were monitored at 7, 14, 21, and 28 days post priming injection to determine the sustainability of the protective effect induced by *A. hydrophila* priming in crab. The enhanced expressions of *Es*ALF-1 and *Es*ALF-3 lasted four weeks, indicating month-long protection in crabs. While the expression levels of the *Es*TLR1 signal remained at a higher level in the first two weeks, and the waning expressions were detected in the last two weeks. The protection of vaccines can fade in months or last a lifetime, and the waning effect of vaccines has also been reported in vertebrates (34–36). The detailed mechanism of waning immunity is complicated, which needs further investigation.

In conclusion, the protective humoral immunity induced by *A. hydrophila* priming was validated in crab *E. sinensis*, and *Es*TLR1-mediated productions of *Es*ALF-3 played an indispensable role in this month-long immune protection. These results provide solid evidence for the molecular basis of enhanced humoral immune protection in crab *E. sinensis* after *A. hydrophila* priming, which would be valuable for the development of disease management strategies in crab aquaculture and understanding the mechanisms of immune priming in invertebrates.

## Data Availability Statement

The original contributions presented in the study are included in the article/**Supplementary Material**. Further inquiries can be directed to the corresponding authors.

## Author Contributions

WW, YL, LW, and LS conceived, designed the experiments, and wrote the manuscript. WW, YL, SF, XS and XL developed the methodology and performed the experiments. WW, YL, XS and QY analyzed the data. XL, QY, and XS contribute to the discussion. All authors contributed to the article and approved the submitted version.

## Funding

This research was supported by National Key R&D Program (2018YFD0900606), grants from the National Science Foundation of China (No. 31802340, 31802324, 31530069), the Fund for Outstanding Talents and Innovative Team of Agricultural Scientific Research in MARA, the Climbing Scholar of Liaoning (to LS), the Research Foundation for Distinguished Professor in Liaoning (to LW, XLYC1902012), the Key R&D Program of Liaoning Province (2017203004 to LS), the Natural Science Foundation of Liaoning, China (20170520056), and the Talented Scholars in Dalian Ocean University.

## Conflict of Interest

The authors declare that the research was conducted in the absence of any commercial or financial relationships that could be construed as a potential conflict of interest.

## Publisher’s Note

All claims expressed in this article are solely those of the authors and do not necessarily represent those of their affiliated organizations, or those of the publisher, the editors and the reviewers. Any product that may be evaluated in this article, or claim that may be made by its manufacturer, is not guaranteed or endorsed by the publisher.

## Supplementary Material

Supplementary Table The expression levels and annotations of total significantly differentially expressed proteins among the three groups (Blank, Ah-12h, Ah-7d).
